# Neutrophil extracellular traps in diseases of the female reproductive organs

**DOI:** 10.3389/fimmu.2025.1589329

**Published:** 2025-05-05

**Authors:** Maria-Laura Morawiec, Robert Kubina, Sebastian Stępień, Marta Smycz-Kubańska, Patrycja Królewska-Daszczyńska, Wioletta Ratajczak-Wrona, Aleksandra Mielczarek-Palacz

**Affiliations:** ^1^ Department of Immunology and Serology, Faculty of Pharmaceutical Sciences in Sosnowiec, Medical University of Silesia in Katowice, Katowice, Poland; ^2^ Silesia LabMed: Centre for Research and Implementation, Medical University of Silesia in Katowice, Katowice, Poland; ^3^ Department of Pathology, Faculty of Pharmaceutical Sciences in Sosnowiec, Medical University of Silesia in Katowice, Katowice, Poland; ^4^ Department of Immunology, Medical University of Bialystok, Białystok, Poland

**Keywords:** NETs, neutrophils, gynecological cancer, gynecological diseases, breast cancer

## Abstract

Neutrophil extracellular traps (NETs) are physiologically released in response to pathogens, serving as a defense mechanism. However, excessive NET production has been implicated in various pathological conditions, including diseases of the female reproductive system. Recent studies highlight the significant role of neutrophils and NETs in cancer pathogenesis. Overproduction of NETs creates sites for tumor cell adhesion, promoting tumor cell proliferation, immune escape, and tumor progression. NET formation is associated with many diseases, including cancers of the female reproductive organs. Detection of NETs can be used as a prognostic tool for patients with diseases characterized by higher rates of NETs formation, such as cancer. In order to use NETs in diagnosis, it is possible to determine them directly or to determine NET components: extracellular DNA, citrullinated histones, NE or MPO. This review explores the role of neutrophils and NETs in the pathogenesis, diagnosis and treatment of breast, ovarian, cervical and endometrial cancer, premature lapse of ovarian function, cervicitis, endometriosis, pregnancy and pregnancy-related diseases.

## Graphical Abstract

NETs in diseases of the female reproductive organs. The figure presents the described diseases divided into cancerous: breast, ovarian, cervical and endometrial cancer and non-cancerous ones: premature ovarian failure, cervicitis, endometriosis, pregnancy and pregnancy-related diseases. All the figures presented in the paper were created in https://BioRender.com.

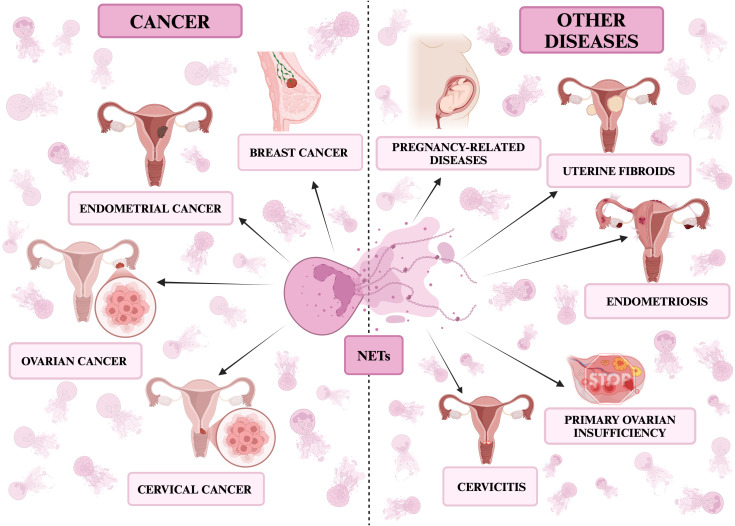

## 1 Introduction

### 1.1 Neutrophils

**Figure 1:**
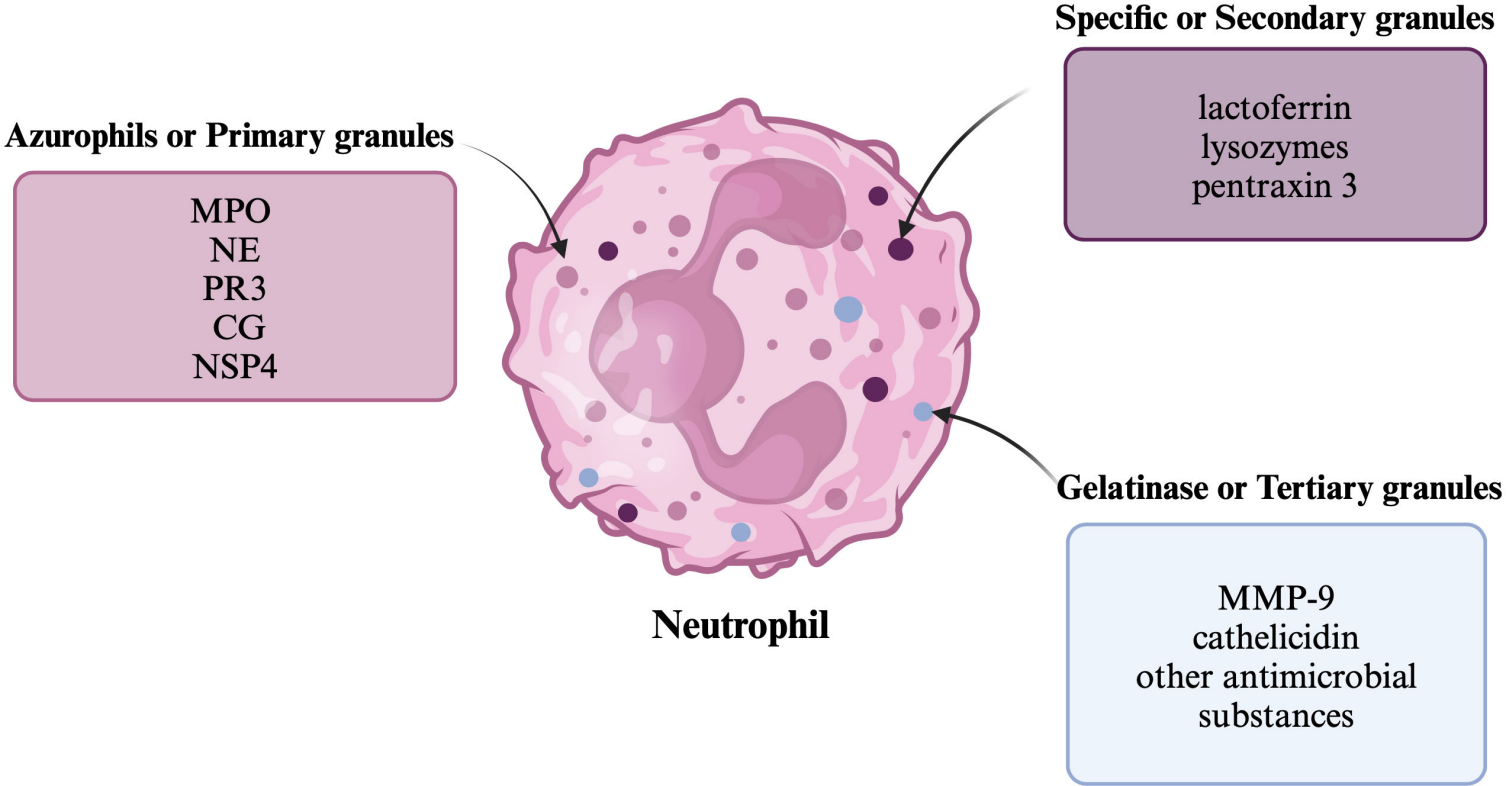
Neutrophil granules The figure shows a neutrophil and the division of its granules, along with examples of substances that belong to them.

Neutrophils are the main, physiologically most abundant leukocyte population in peripheral blood in adults (50-70%), where they are present for about 12 hours (1–3). The neutrophil population can be divided into three main groups: bone marrow reserve, circulating and located in peripheral tissues (4). They are produced in the bone marrow within hematopoietic cords surrounded by venous sinusoids, while they arise from stem cells that proliferate and differentiate into mature neutrophils equipped with granules (1, 5). Granules can be divided into primary (azurophils), secondary (specific) and tertiary (gelatinase) (6). Primary granules consist mainly of myeloperoxidase (MPO) and neutrophil serine proteases (NSPs) (6). NSPs include neutrophil elastase (NE), proteinase 3 (PR3), cathepsin G and neutrophil serine protease-4 (NSP4) (7). Secondary granules contain lactoferrin, lysozymes, and pentraxin 3, while tertiary granules consist of matrix metalloproteinase-9 (MMP-9) and antimicrobial substances, including cathelicidin (6, 8), which have been shown on the **Figure 1**.

**Figure 2:**
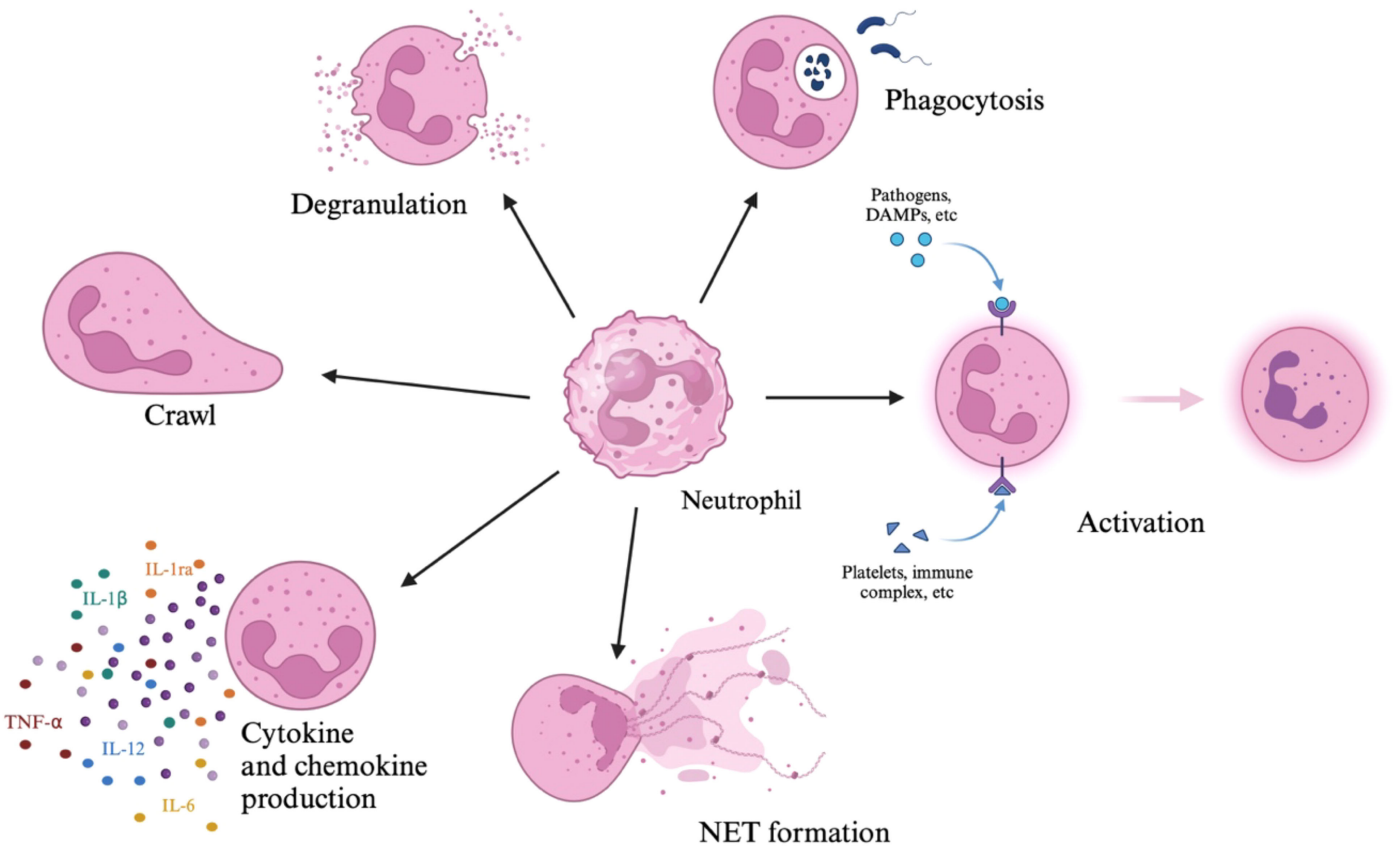
Neutrophils’ functions. The figure shows neutrophil functions, which include degranulation, phagocytosis, activation, formation of NETs, production of chemokines and cytokines, and crawling.

The activity of neutrophils is the basis for the operation of the innate immune response, as they are among the first cells of the immune system to respond to pathogens (including bacteria, fungi and protozoa) (9, 10). The life cycle of neutrophils and their maturation is associated with their acquisition of functions, and as the main effector cells of the immune system, they have numerous capabilities to combat pathogens: phagocytosis, migration, production of reactive oxygen species (ROS), degranulation and, consequently, release of cytotoxic granule components and recruitment of other immune cells (1). Neutrophils can shape the inflammatory and immune response through production of cytokines and chemokines, including, among others: tumor necrosis factor alpha (TNF-α), interleukin 1β (IL-1β), interleukin-1 receptor antagonist (IL-1Ra), interleukin 6 (IL-6), and interleukin 8 (IL-8) (11–13). They also have the ability to form neutrophil extracellular traps (NETs) (13). The neutrophil functions are shown in **Figure 2**.

### 1.2 Neutrophils in diseases of the female reproductive organs

In recent years, attention has been drawn to the significant role of neutrophils, not only in fighting pathogens but also in the pathomechanism of cancer. It has been shown that in addition to macrophages, subpopulations of T lymphocytes, B lymphocytes, dendritic cells or Natural Killer cells (NK cells), tumor-associated neutrophils (TAN) are an important component of the tumor microenvironment (TME) (14–16).

A study by Fridlender et al. (17) showed the existence of at least two different populations of tumor-associated neutrophils: pro-tumor and anti-tumor. The dichotomous role of neutrophils depends on cytokine signaling and epigenetic modifications and is enabled by signals from tumor cells or cells within the tumor microenvironment (2, 18). Tumor-derived factors and the tumor microenvironment have been shown to have the ability to reprogram neutrophils from an anti-tumor phenotype to a pro-tumor phenotype (19, 20). Tumor-derived cytokines: transforming growth factor-beta (TGF-β), granulocyte colony-stimulating factor (G-CSF), and interferon-beta (IFN-β) are involved in neutrophil polarization (21). G-CSF secreted by tumor cells can alter the hematopoietic function of the bone marrow and promote neutrophil differentiation toward the N2 phenotype (20). TGF-β activates the tumor-promoting neutrophil program, i.e., pro-tumor polarization, while IFN-β promotes the opposite process, i.e., anti-tumor polarization (21).

The N1, “anti-tumor” neutrophil phenotype promotes tumor suppression (20). Studies indicate that in the pre-metastatic niche, factors such as TGF-β, for instance, hinder the emergence of the N1 phenotype, thereby preventing extensive killing of tumor cells (20). Antitumor neutrophils can directly kill tumor cells by releasing ROS and reactive nitrogen species (RNS) (14). Neutrophils can recruit other immune cells to the TME, including M1 macrophages with pro-inflammatory and anti-tumor activity (14, 22, 23). Neutrophils are able to inhibit metastasis through cytotoxicity towards tumor cells in the circulation or in the pre-metastatic niche and by stimulating T cells proliferation (14, 22). They also have the ability to present antigens to T cells and to produce interferon- gamma (IFN-γ) (14).

**Figure 3:**
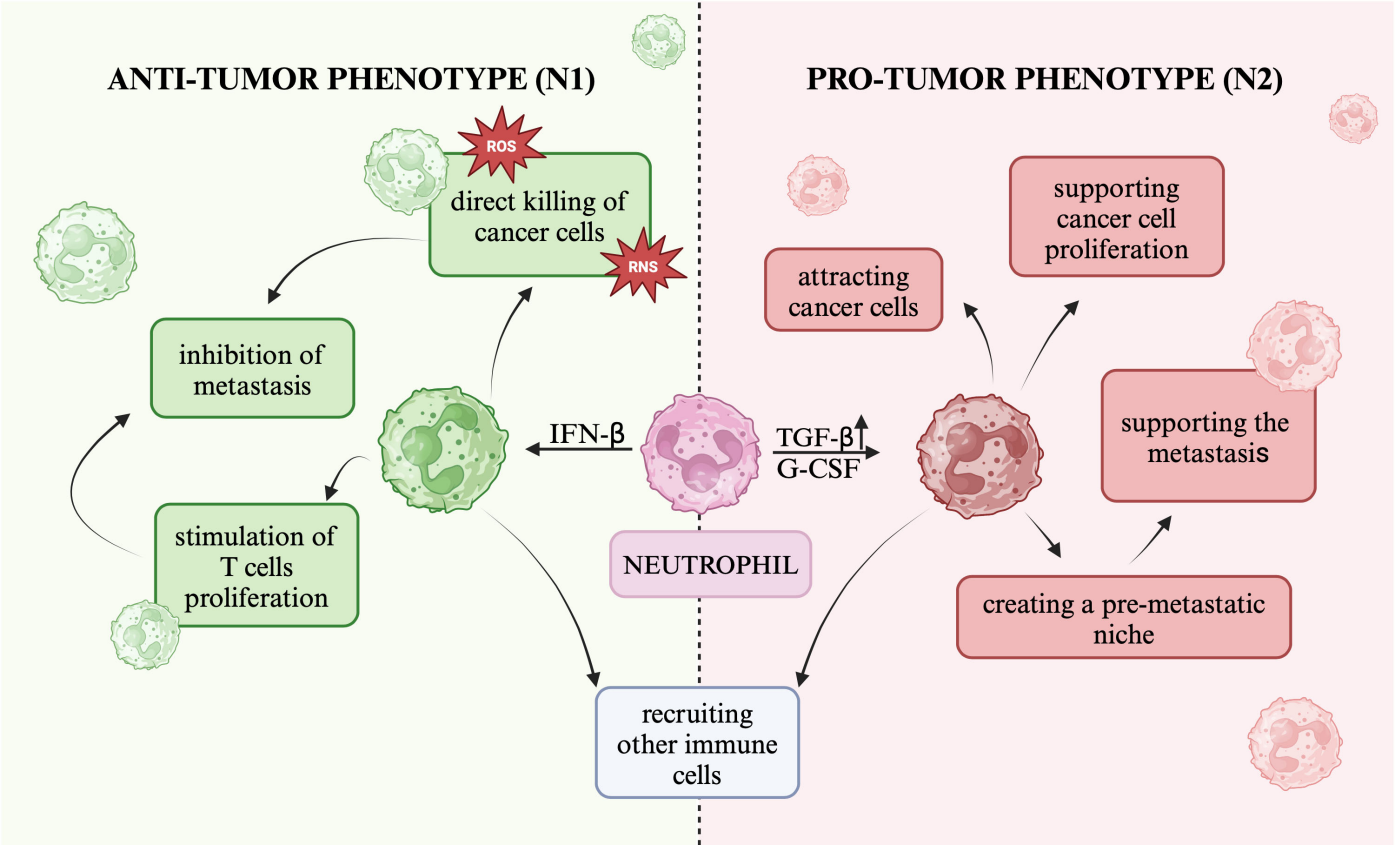
The role of pro-tumor and anti-tumor neutrophils The figure presents two neutrophil phenotypes: the anti-tumor phenotype (N1) and the pro-tumor phenotype (N2), and the role they may play in carcinogenesis.

In the presence of cytokines such as TGF-β, available in high concentrations at the primary tumor site, neutrophils acquire a pro-tumor phenotype - N2 (24). The neutrophil N2 phenotype is shaped by the premetastatic microenvironment and may promote tumor cell dissemination and progression (20). Protumorigenic neutrophils actively support metastasis through various mechanisms, including the formation of a premetastatic niche, attraction of tumor cells and direct promotion of tumor cell proliferation (22). The influence on the immunosuppressive environment in the pre-metastatic niche is related to the ability to secrete arginase to degrade arginine, which is crucial for the effectiveness of tumor killing by T cells (20). Protumor neutrophils can release MMP-9, which promotes angiogenesis and tumor cell proliferation and can suppress NK cells function (14). Neutrophils recruit other immune cells that can have dual effects on the TME, for example, anti-inflammatory and pro-tumor M2 macrophages and regulatory T cells (14, 23). The role of pro-tumor and anti-tumor neutrophils is shown in **Figure 3**.

The discovered role of neutrophils in the following diseases is described below: breast, ovarian, cervical cancer, corpus uteri cancers, premature ovarian failure and endometriosis.

#### 1.2.1 Breast cancer

Yin et al. (25) detected TAN-related genes and investigated their association with breast cancer. Patients with these genes showed tumor immunosuppression and adverse therapeutic effects (25). TANs correlate with poor breast cancer prognosis (25, 26). High TAN density correlates with unfavorable prognostic factors in breast cancer such as: large tumor size, type and unfavorable histological grade, high rate of lymph node metastasis, advanced stage of disease, breast cancer subtype and selected mutations: MAP3K1, ERBB2 and TP53 (26). TANs are able to secrete oncostatin M, which promotes the secretion of VEGF in the environment of human breast cancer cells and increases their ability to invade (27). G-CSF secreted by tumor cells stimulates pro-tumorigenic neutrophils functions in invasive breast cancer (21). Breast cancer patients with circulating tumor cell and neutrophil aggregates showed worse progression-free survival than patients without such aggregates (21). Improved survival has been demonstrated in patients with breast cancer and other cancers who experience neutropenia during chemotherapy, which may be due to a reduction in pro-metastatic neutrophils (28). In luminal A and luminal B subtypes, researchers found no association between neutrophil-to-lymphocyte ratio (NLR) and overall survival in breast cancer patients (29). However, a correlation was detected in the analyses of HER2-positive breast cancer and triple negative breast cancer (TNBC) (29). NLR is currently used as a predictor of overall mortality and cancer-free survival (30). However, it should be noted that the increase in the number of neutrophils is not specific to the cancer process, it is also observed in infections and inflammation (31, 32). NLR therefore reflects well the inflammation that plays an important role in the progression of some cancers and the formation of metastases (29).

#### 1.2.2 Ovarian cancer

In ovarian cancer, it has been shown that neutrophils can have a deregulating effect on the immune system, potentially contributing to the progression and metastatic potential of cancer cells (33). Elevated NLR ratio in ovarian cancer patients before treatment may be a predictor of poor disease prognosis (31). NLR was significantly higher in patients with ovarian cancer compared to patients with benign ovarian tumor, other gynecological diseases and healthy individuals (34–36). Elevated TAN levels are associated with poor prognosis and immune tolerance in ovarian cancer (34). Mayer et al. (35) demonstrated that neutrophils in the ovarian cancer microenvironment can shift tumor cells toward a mesenchymal and migratory phenotype. Interestingly, this effect was observed after incubation of cancer cells with neutrophil elastase, which is also a component of NETs (35).

#### 1.2.3 Cervical cancer

In cervical cancer, neutrophils have been shown to possibly contribute to the progression and metastatic potential of cancer cells (33). A significant association has been demonstrated between neutrophilia and advanced cervical cancer (37). More than 10% of cervical cancer patients experienced tumor-related leukocytosis (TRL) detected at initial diagnosis (38). Carus et al. (39) demonstrated that the number of TAN is an independent prognostic factor for short recurrence-free survival in localized cervical cancer. Elevated NLR was associated with worse overall patient survival and shorter progression-free survival in patients with cervical cancer (40). NLR can therefore be used as a prognostic indicator in patients with cervical cancer (41). However, its prognostic value may be higher in locally advanced and/or advanced cervical cancer compared to patients with early stage disease (33). High density of infiltrating neutrophils in cervical cancer tissues was associated with poor prognosis (42).

#### 1.2.4 Other corpus uteri cancers

Srisutha et al. (43) showed that in patients diagnosed with uterine leiomyosarcoma, the NLR was significantly higher than in patients diagnosed with uterine leiomyoma. NLR is therefore an effective marker of prediction the presence of uterine leiomyosarcoma in patients preoperatively diagnosed with a uterine tumor (43).

#### 1.2.5 Premature ovarian insufficiency

Premature ovarian insufficiency (POI) is caused by a decline in ovarian function due to premature depletion of follicles (44). The NLR ratio was statistically higher in the POI group compared to the control group and also correlated with follicle-stimulating hormone (FSH) and Anti-Müllerian Hormone (AMH) (45). Ilhan et al. (45) proved that it can be a marker for POI diagnosis.

#### 1.2.6 Endometriosis

It has been shown that in the circulatory system and peritoneal fluid of patients with endometriosis there is an increased number of neutrophils and cytokines released by them, which promotes endometrial cell proliferation and invasion (46–48). Comparison of NLR values ​​with CA125, endometriosis stage and painful menstruation, after taking into account previous therapy, did not show any significant association (46). However, an association between NLR and chronic pelvic pain has been demonstrated (46).

## 2 NETs

NETs are web-like filamentous extracellular structures released by neutrophils in response to pathogens such as bacteria, fungi, protozoa (2, 49, 50). These nets effectively capture and kill the mentioned pathogens, thereby minimizing tissue damage (49). The occurrence of this phenomenon was first noted by Takei et al. (50) in 1996 and described and named by Brinkmann et al. (51) in 2004. The formation of extracellular traps has also been observed in other cells, not only neutrophils, such as macrophages (52), monocytes (53), mast cells (54), eosinophils (55), plasmacytoid dendritic cells (56), basophils (57), B cells and T cells (58).

### 2.1 NETs structure

NETs are three-dimensional networks composed of deoxyribonucleic acid (DNA) fibers with a diameter of 15–17 nm and protein substances, granule components, with a diameter of about 25 nm, including histones H1-H4, proteases and other cytotoxic and highly inflammatory compounds, including: MPO, NE, lactoferrin, defensins, lysozyme C, azurocidin, cathelicidin, CG, calprotectin, pentraxin 3, MMP-9, NSP, gelatinase (1, 2, 6, 59–61).

**Figure 4:**
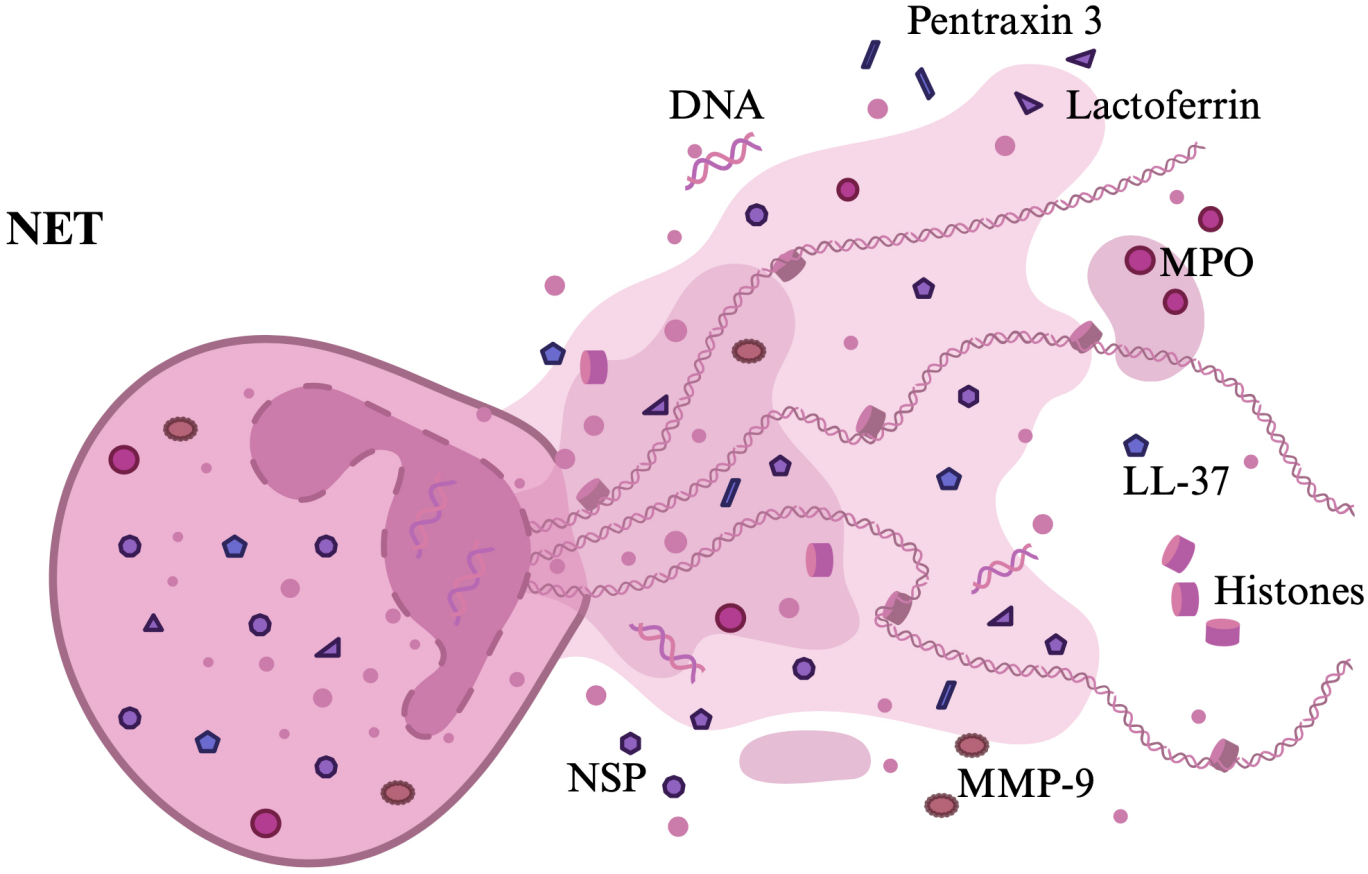
NET structure. The figure presents the NET structure with the most important components that have been described.

The adhesive properties of nucleic acids, as well as the action of histones and neutrophil enzymes in the extracellular environment make NETs contribute to the host defense against various microorganisms (60). Trapping and inactivation of pathogens by the network is possible due to the high local concentration of granule-derived substances and histones, which have antibacterial activity and are the most abundant proteins in NETs (3, 61–63). Proteolytic enzymes released from the granules have the ability to degrade bacterial virulence factors (63). MPO plays an important role in defense against bacteria, viruses and fungi by converting hydrogen peroxidase to hypochlorous acid (61). MPO activity can also cause damage to adjacent tissues and thus contribute to the pathogenesis of various inflammatory diseases (61). NE is a neutrophil-specific serine protease that degrades virulence factors and neutralizes bacteria (64). Lactoferrin has the ability to move from the cytoplasm to the plasma membrane and inhibit the release of NETs (65). Thus, it may act as an internal inhibitor of NETs release into the bloodstream (65). Cathelicidin, or LL-37, is an antimicrobial peptide that also has immunomodulatory properties (52, 66). Each NET protein has a function, for example, calprotectin is responsible for the antifungal properties of NETs (67). Pentraxin 3 protects against extracellular histone-mediated cytotoxicity, has an antibacterial role, and likely mitigates the harmful effects of overactivated NETs (68). MMP-9 has the ability to degrade extracellular matrix components (69). NSPs play a key role in the antimicrobial response (7). They are located in granules tightly bound to proteoglycans, which inactivate them, while they become active only after being released into the phagocytic vacuole (7). The main role of NSP associated with NETs is their induction (7). The structure of NET is graphically presented in **Figure 4**.

### 2.2 NETs formation

NETs formation involves chromatin decondensation by neutrophils in a process that requires neutrophil activation, ROS, NE, MPO activity and histone citrullination (1, 70, 71). Neutrophils contain relatively few mitochondria and obtain most of their energy from glycolysis (72). NETs formation is glucose-dependent, and to a lesser extent glutamine-dependent (72). Lipid metabolism is also involved in the formation of NETs (70). Requirements for macro- and micronutrients needed to form NETs are still ambiguous (73). NETs formation is influenced by certain trace elements, primarily calcium ion, but also zinc, phosphorus, sulfur, iron and copper (74, 75). Some of these elements were also found in the network itself (76). Most neutrophil DNA is transcriptionally inactive and condensed to heterochromatin in the cell nucleus (63). DNA is wrapped around histones, forming nucleosomes and further organized into chromatin (63). Peptidyl arginine deiminase 4 (PAD4) mediates heterochromatin decondensation by catalyzing the conversion of histone arginines to citrulline, reducing the strong positive charge of histones and weakening histone-DNA binding (65, 77). ROS can also activate PAD4, which is found in high concentrations in neutrophils (2, 75). There is also an increase in intracellular Ca^2+^, as these cations act as cofactors for PAD4, a nuclear enzyme that promotes the described histone deamination (59). ROS activate key proteins involved in different parts of the process, stimulate the release of MPO and NE from azurophilic granules into the cytosol, and from there into the nucleus, where elastase digests histones, assisted by MPO (2, 78–80). NE degrades the actin cytoskeleton, impeding the ability to move and phagocytose, and promotes chromatin decondensation (1, 59). Generation of ROS, mediated by NADPH oxidase 2 (NOX2) is the critical step in chromatin decondensation (81). ROS promote the gradual separation and loss of the nuclear membrane, which disintegrates into small individual vesicles (59). The chromatin then disperses into the cytoplasm, where it mixes with cytoplasmic proteins and granules toxins (2, 61). Finally, DNA structures with histones and proteins are released into the extracellular space after the integrity of the cytoplasmic membrane is lost, in a process involving the polymerization of gasdermine D, which participates in the formation of membrane pores (1, 82).

**Figure 5:**
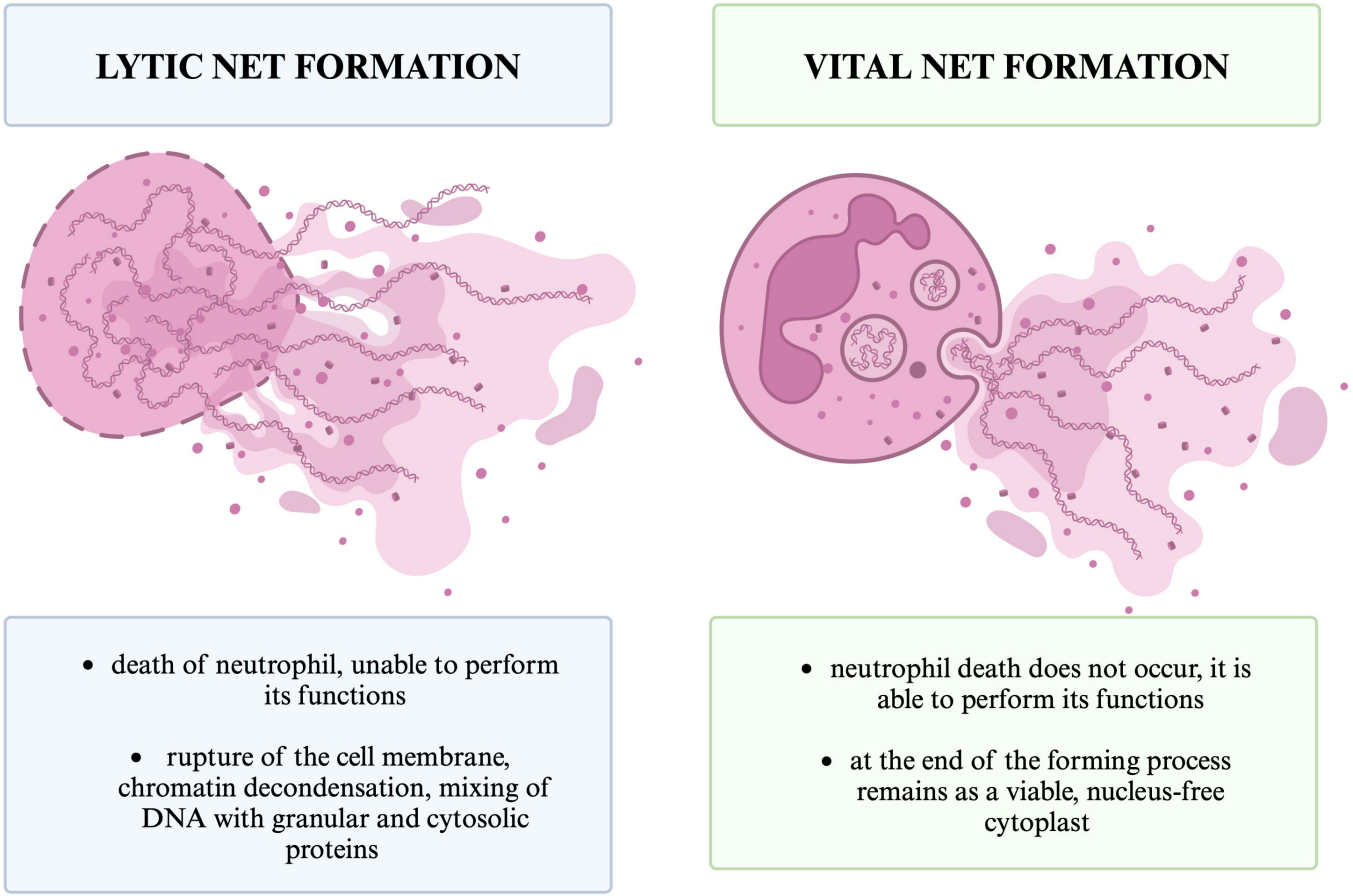
Different types of NET formation. The figure shows the two basic types of NETs formation that we distinguish, lytic and vital, and the basic differences between them.

NET formation usually leads to lytic neutrophil death (also referred to as suicide), termed NETosis (2-3). Non-lytic forms of NET formation; vital and mitochondrial, have also been observed (2-3). Different types of NET formation are shown in **Figure 5**. Different types of NET formation can be defined taking into account the origin of released DNA, the inducing stimuli, the morphological changes undergone by the neutrophil and its viability after the process (3). It should also be noted that only a small fraction of circulating neutrophils form NETs after stimulation (24).

Lytic NET formation involves chromatin decondensation and mixing of DNA with granular and cytosolic proteins within the neutrophil, which leads to rupture of the cell membrane and neutrophil death (79). The second mechanism is vital NET formation, that do not lead to neutrophil death (70). During vital or non-lytic NET formation, neutrophil degranulation and chromatin release leads to extracellular NET formation and leaves a viable, nucleus-free cytoplast that can perform neutrophil functions such as crawling and phagocytosis (3, 82). We can also distinguish mitochondrial NET formation, which leads to NETs composed of mitochondrial DNA released by viable cells (70). In 2018, the Nomenclature Committee on Cell Death (NCCD) recommended replacing the term “NETosis” with “NETs” or “NET formation” to include the occurrence of the mechanism also in the absence of cell death (82, 83, 84).

The first described pathway, referred to as suicidal or lytic NETosis, occurs after stimulation by triggers such as phorbol myristate acetate (PMA), Gram-negative and Gram-positive bacteria, lipopolysaccharide (LPS), antibodies, cholesterol crystals, high-mobility group box 1 (HMGB1), proinflammatory cytokines, including TNFα, IL-1β, and IL-8 (2, 49, 61, 63, 85–87). Microorganisms or PMA are the most potent triggers of NETosis, as they induce the process in about 30% of the population (85). The variety of factors that activate the process suggests the existence of different pathways for NETosis activation (85).

The mechanism of lytic NETosis includes stimulation of protein kinase C (PKC) and Raf-MEK-ERK signaling, activation of NADPH oxidase (NOX) and production of ROS (2). It has also been suggested that NETotic cell death results from a signaling pathway involving the Raf-1 proto-oncogene, serine/threonine kinase (RAF1), mitogen-activated protein kinase kinase (MAP2K) and extracellular signal-regulated kinases (ERK2) (84). Also, activation of Rho GTPases leads to increased NET formation in response to extracellular cold-inducible RNA-binding protein (eCIRP) stimulation (70). eCIRP plays a role by acting as a Danger/Damage Associated Molecular Pattern (DAMP), which links to inflammatory diseases, and has the ability to induce an inflammatory response in macrophages, neutrophils, lymphocytes, and dendritic cells (86).

3 to 8 hours after activation, neutrophils initiate the NETosis mechanism in response to triggers and activate PKC, which leads to calcium fluxes in the cell and activation of the NADPH oxidase (NOX) signaling cascade (60, 88). There is a large amount of NADPH on the cell membrane and phagosome membrane of neutrophils (73). Activation of the signaling cascade results in NADPH-dependent production of ROS, the release of which is crucial to the process of NETosis (60, 88–90). The produced hydrogen peroxide is used by MPO to produce hypochlorous acid and other oxidants (91). ROS are responsible for the oxidative burst that kills the phagocytosed organism in the phagolysosome (85). This is the first discovered and best described mechanism of NET formation (2, 60).

Raf-MEK-ERK pathway is involved in NET formation through activation of NADPH oxidase and up-regulation of anti-apoptotic proteins (88). In particular, it has been shown that it can modulate NADPH oxidase and also affect the expression of the anti-apoptotic protein Mcl-1, which inhibits apoptosis and activates ROS to promote NETosis (2). For example, Entamoeba histolytica, which causes amoebiasis, activates a signaling pathway to induce NET formation that involves Raf-MEK-ERK, but is independent of PKC, transforming growth factor-β-activated kinase 1 (TAK1) and ROS (89). The release of NETs induced by parasites, for example *Toxoplasma gondii*, involves the MEK-ERK pathway (92). After PMA stimulation, PKC activity increased to allow endoplasmic calcium to enter the cytoplasm, which then phosphorylates NOX2, driving ROS production (81). Phagocytic NOX2 is a highly regulated membrane-bound multiprotein complex that produces large amounts of superoxide anion radical, which leads to an oxidative burst (93). Similar to PMA, NOX2 phosphorylation was mediated by LPS stimulation via the c-Jun N-terminal kinase (JNK) pathway (94).

NADPH oxidase-independent NET formation is a rapid calcium-activated pathway (81). It can be stimulated, for example, by mitochondrial DNA (95). Even if NADPH oxidase is not essential for NET formation, ROS generation is required (81).

Vital NETosis owes its name to the maintenance of intact neutrophil cell membranes, which enables them to maintain physiological functions (2). It’s independent of NADPH, ROS production and the Raf/MERK/ERK pathway (59, 96). This process is faster than lytic NETosis, as it occurs within 5-60 minutes (59, 96). Vital NET formation can be induced by *Staphylococcus aureus* within minutes via complement receptors and Toll-like receptor 2 (TLR2) ligand (70). Alternatively, it can be induced by activation of Toll-like receptor 4 (TLR4) by *Escherichia coli* (2). TLR4 found on platelets also contributes to this process, as do DAMPs, which enhance ongoing immune responses (2, 97, 98). PAD4 is activated, which induces chromatin decondensation (2). DNA-containing nuclei are disassembled without disturbing the cytoplasmic membrane, and decondensed chromatin is transported through vesicles to expel DNA (2). Once NETs are released, neutrophils remain vital and capable of phagocytosis and chemotaxis (99).

The release of mitochondrial DNA is another type of vital NETosis, which is ROS-dependent and produced after stimulation with granulocyte-macrophage colony-stimulating factor (GM-CSF) and LPS (24). Yousefi et al. (97) showed that GM-CSF stimulation and subsequent short-term stimulation of TLR4 or complement factor 5a (C5a) receptor enabled viable neutrophils to generate NETs. They also proved that NETs formed by living cells contain mitochondrial, not nuclear DNA (97). The association between mitochondrial NETs and autoimmune diseases was shown by Lood et al. (100). NETs may therefore consist of mitochondrial DNA (101). It is still unclear whether the amount of mitochondrial DNA is sufficient for effective NET formation, given the reduced number of mitochondria in neutrophils (101).

### 2.3 NETs degradation

Some bacteria, for example Group A Streptococcus, have evolved and produce an extracellular DNase that degrades NET by cleaving DNA strands and promotes virulence (102–105). Beiter et al. (102) showed that endonuclease enables pneumococci to degrade NETs. Kolaczkowska et al. (103) showed that DNase dissolves DNA without affecting histones and neutrophil elastase derived from the net.

### 2.4 The positive and negative aspects of NETs

Although the formation of NETs is directly related to neutrophils, which have the ability to phagocytose and kill microorganisms, and NETs have the ability to capture, prevent the spread and neutralize pathogens, their presence and activity has also been associated with various diseases, thrombosis, fibrosis, and delayed wound healing (106–109). Excessive and uncontrolled release of NETs can cause adverse effects on the course of some diseases, such as exacerbation of inflammation and tissue damage (88, 110). The role of neutrophil extracellular traps has been described in the pathogenesis of numerous diseases, for example autoimmune diseases such as chronic granulomatous disease, lupus erythematosus and rheumatoid arthritis (107). The indirect role of NETs mechanism has also been described in the pathogenesis of diabetes and atherosclerotic lesions (107). Neutrophils are rarely found in the brain due to the presence of a selective blood-brain barrier (82). In cases of its damage, for example in traumatic brain injuries, spinal cord injuries, bacterial meningitis, ischemic stroke, glioma, multiple sclerosis and Alzheimer’s disease, the presence of neutrophils and their extracellular traps has been observed (82). The presence of NETs has also been described in other pathological processes, such as sepsis, coagulation disorders, pre-eclampsia, obesity, cystic fibrosis, COVID-19, periodontitis, malaria, and tuberculosis (110–115).

**Figure 6:**
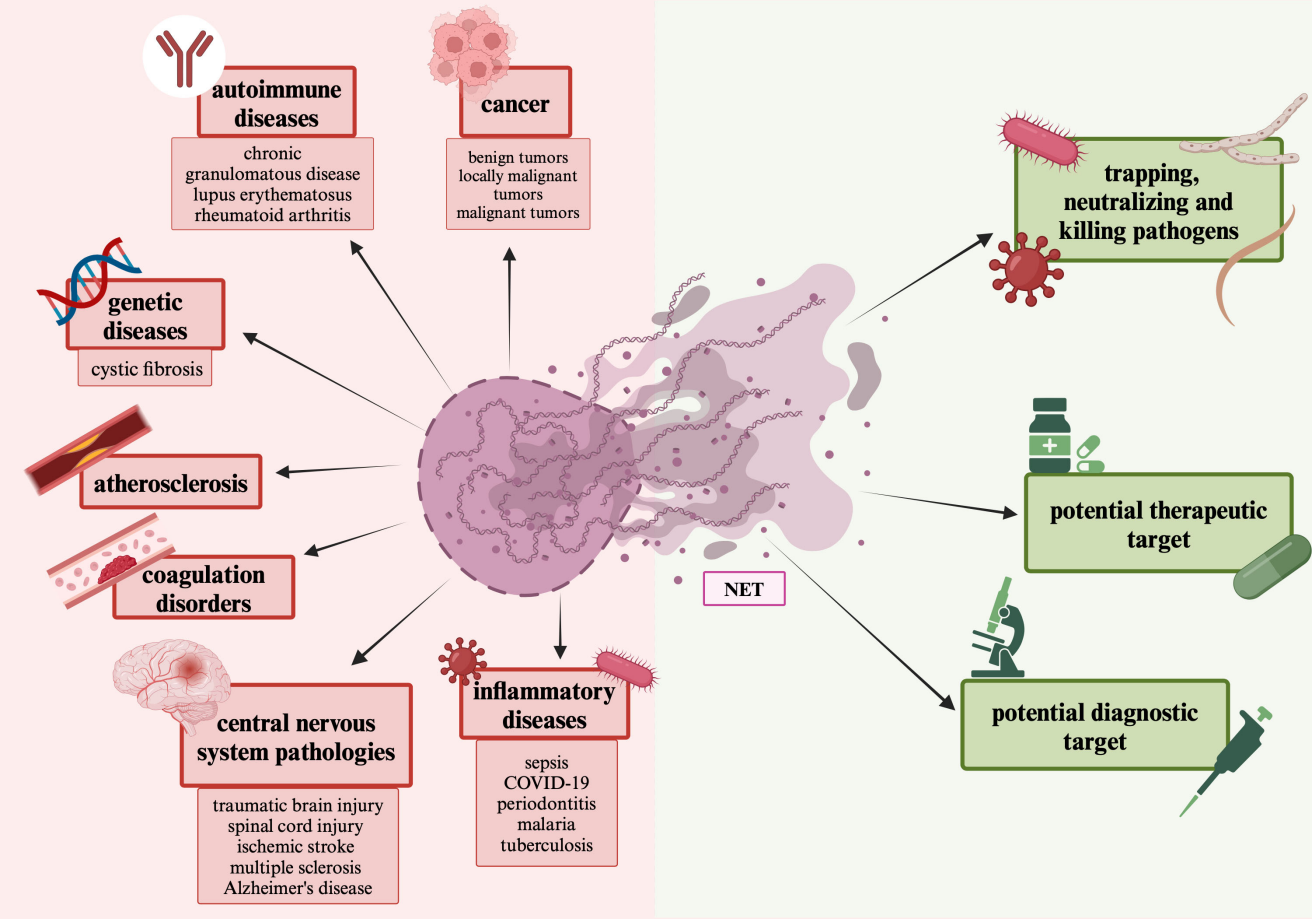
The positive and negative aspects of NETs. The figure depicts the positive aspects associated with NETs: trapping and neutralizing pathogens, their diagnostic and therapeutic potential, and the negative aspects associated with NETs, participation in various diseases, presented with their segregation.

NETs can also serve as a therapeutic and diagnostic target. The presence and formation of NETs accompanies many diseases, so preventing their formation may be a potential therapeutic strategy (96). NETs detection can be used as a prognostic tool for patients with diseases characterized by a higher rate of NETs formation, for example cancer (116). In order to use NETs in the diagnosis, it’s possible to either determine them directly or determine the components of NETs: extracellular DNA (cell free DNA, cfDNA), citrullinated histones (citH3), NE or MPO (116). For example, the amount of cfDNA in the serum of patients with different types of cancer and patients with metastases is higher than in healthy individuals (117–120). Cell-free DNA does not come specifically from neutrophils, but also from other cells that form extracellular traps (117). The positive and negative aspects of NETs are shown in **Figure 6**.

### 2.5 The pro-cancer role of NETs

**Figure 7:**
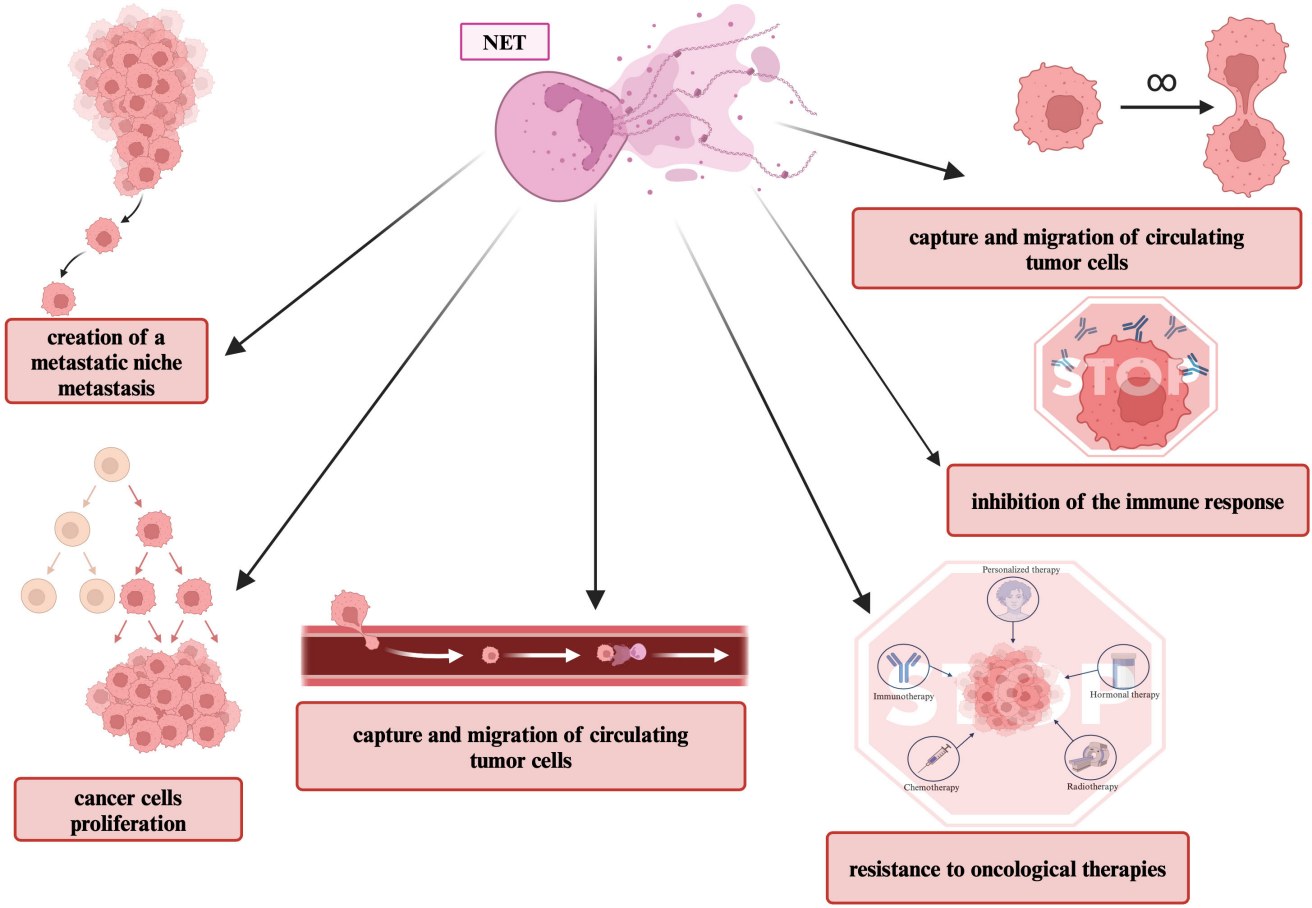
Pro-cancer role of NETs. This figure shows pro-tumor features that are associated with NETS formation.

It has recently been known that neutrophils and NETs are involved in the pathogenesis and progression of cancer (78). The presence of NETs has been associated with poor prognosis in cancer patients (121). As the tumor grows, more NETs appear in the TME, which correlates with increased tumor cell proliferation (122). There is an increasing number of studies emerging describing the pro-tumor effects of NET formation (78). The occurrence of NETs has been described in both primary human tumors and metastases (123). The role of NETs in metastasis has already been widely described (111, 124–127). Several pro-neutrophil tumorigenic strategies involving the NET mechanism have been documented, including promotion of proliferation, uptake and migration of circulating tumor cells, formation of a pre-metastatic niche, increased tumor cell survival, suppression of the immune response, and resistance to immunotherapy and other cancer treatments (2, 123, 126). The pro-cancer role of NETs is shown in **Figure 7**.

NETs induce epithelial-to-mesenchymal transition (EMT) in cancer cells and promote angiogenesis thereby increasing tumor invasiveness (113, 128). NETs may cause changes in the immune system’s interactions with cancer cells (111). Within the TME, NETs physically protect cancer cells from cytotoxic T cells and NK cells by surrounding the cancer cells (126). Teijeira et al. (129) demonstrated that CXCR1 and CXCR2 receptor agonists, frequently produced in tumors, are the main mediators of cancer-promoted NETs formation. Components of the pre-metastatic niche also accelerate the generation of NETs, with IL-8, HMGB1 and G-CSF derived from tumor cells being key initiating factors (20, 130).

NETs-related proteins are also involved in tumor progression (131). NE activates dormant tumor cells, promotes proliferation, tumor cell invasion and distant metastasis, induces EMT, and alters the tumor microenvironment (132, 133). Wada et al. (132) showed that NE can also increase the levels of transforming growth factor alpha (TGF-α), vascular endothelial growth factor (VEGF) and platelet-derived growth factor (PDGF) in cultured cancer cells. NE has the ability to degrade extracellular matrix, hydrolyze fibronectin, proteoglycans, collagen type IV and other proteins (20). NE also has the ability to induce phosphatidylinositol 3-kinase (PI3K) pathway in cancer cells (78). Induction of the PI3K signaling pathway promotes cancer cell proliferation, migration and is also associated with metastasis and angiogenesis (133). MMP-9 has been linked to each stage of carcinogenesis (69). It has been shown that cathepsin G can also facilitate angiogenesis and tumor cell migration (134). Cancer cells can aggregate in blood vessels and form tumor emboli at distant sites (134). Cathepsin G has been shown to facilitate tumor aggregate formation in human breast cancer cells (135). This aggregation was mediated by E-cadherin-mediated intracellular adhesion (135). It has also been proven that inhibition of cathepsin G reduced cancer cell aggregation (134). In addition to NETs components, their special structure also has pro-cancer properties (134). Circulating tumor cells (CTCs) are a major contributor to cancer metastasis (134). Due to their structure and viscosity, NETs are able to trap CTCs, thus promoting the adhesion of tumor cells to distant organs (59, 136).

NETs promote inflammation, which develops a positive feedback loop: NETs released into the circulation damage endothelial cells, which increases inflammation, causing activation of platelets and other neutrophils, which can trigger further release of NETs (137). Cancer-related platelet activation facilitates tumor progression and metastasis and enables EMT (78). Cancer-related thrombosis (CAT) is a thrombotic event that occurs as a complication of cancer treatment (136). The main risk factors for venous thromboembolism (VTE) in cancer patients also include the type and location of the tumor (136). The risk of VTE depending on the type of cancer can be divided into three groups: high risk (gynecological cancers), medium risk and low risk (breast cancers) (136). Various cancers have been shown to be capable of predisposing circulating neutrophils to produce NETs that can cause systemic thrombosis and embolism (138). Also, cfDNA can enable platelet adhesion and trigger platelet activation, thereby activating blood coagulation (139). NETs also trigger thrombosis by trapping circulating platelets and extracellular vesicles (136). Intravascular NETs occur in greater numbers in cancer patients and are associated with a higher incidence of venous thrombosis compared to patients without cancer (140).

### 2.6 The anti-cancer role of NETs

Despite abundant evidence of a pro-tumor effect of NETs, it has also been shown that the components released by NETs: MPO and histones, directly inactivate cancer cells (111). The anti-cancer components released by NETs also include NE, which in some cancers has the ability to selectively kill cancer cells and attenuate carcinogenesis (132, 141). Catalytically active neutrophil elastase (ELANE) has been identified as the major antitumor protein released by neutrophils (142). MMP-9 promotes tumor development and progression in most cases, and in some specific cases may also play a suppressive role in tumor progression (143). Histones, another important component of NET, are able to damage epithelial cells and consequently damage the blood vessels feeding the tumor (134).

NETs influence cancer immunoediting and may therefore also support antitumor immune responses (141). Depending on their phenotype, neutrophils can kill disseminated cancer cells (22). Entrapment of cancer cells by NETs provides closer contact, which aids by promoting direct killing by activated neutrophils (22). NETs may support metastatic processes, but they may also be involved in neutrophil anti-metastatic responses, as they can be used to lower the threshold for T cell activation (22). This increases the response of T cells to specific antigens (22). From the perspective of antitumor immunity, NETs may therefore inhibit tumor growth by activating the immune system (144). In addition, NETs can modulate immune responses by activating plasmacytoid dendritic cells (pDC) (49).

## 3 NETs in diseases of female reproductive organs

In this review, we will discuss the role of neutrophils and NETs in the pathogenesis, diagnosis and treatment of breast, ovarian, cervical and endometrial cancer, premature ovarian failure, cervicitis, endometriosis, pregnancy and pregnancy-related diseases.

### 3.1 Breast cancer

The molecular classification of breast cancer is based on the expression of estrogen receptor/progesterone receptor (ER/PR), human epidermal growth factor receptor 2 (HER2) and the proliferative antigen index Ki-67 (145). Based on the expression or lack of expression of the above-mentioned receptors, the following molecular subtypes of breast cancer can be distinguished: luminal A, luminal B, non-luminal and TNBC (145). TAN has been detected in most TNBC tumors, the most deadly subtype of breast cancer (21, 146, 147). Compared to other types of breast cancer, it is characterized by high invasiveness, high risk of recurrence and distant rate of metastasis (148).

In breast cancer, data indicate that NETs are involved in various stages of tumor development, particularly in the metastatic phase (149). High levels of NETs correlated with disease progression, metastasis and vascular complications such as venous thromboembolism (137).

Tumor-secreted protease cathepsin C (CTSC) promotes breast-to-lung metastasis by regulating neutrophil recruitment and NETs formation, as studied on cell lines by Xiao et al. (150). NETs also have the ability to activate tumor growth by degrading thrombospondin-1 (TSP-1), which enables tumor cell colonization (150). Martins-Cardoso et al. (151) evaluated the ability of isolated NETs to modulate the phenotype of prometastatic human breast cancer cells. Incubation of isolated NETs with a luminal cell line changed the epithelial morphology to a mesenchymal phenotype, whose cells showed increased migratory properties (151). The researchers also showed that NETs regulated gene expression of several factors associated with the proinflammatory and prometastatic properties of breast cancer cells, including IL-1β, IL-6, IL-8, CXCR1, MMP-2, MMP9, and CD44 (151). The researchers’ results suggest that NETs released in the primary tumor may contribute to the acquisition of metastatic properties during breast cancer progression, and that modulation of NET formation during tumor progression may represent a therapeutic target to reduce metastatic spread (151). NETs and abnormal activation of the NF-κB pathway have been associated with breast cancer progression (152). Zhu et al. (152) showed that PMA-induced NETs promote breast cancer cell progression, and that cancer cell-derived factors: IL-8 and GCS-F, stimulate neutrophils to form NETs. NETs formation correlates with regulatory T-cell infiltration in breast cancer (153). This infiltration is the result of multiple steps that begin with collagen, which increases the expression of DDR1, a discoidin domain receptor (DDR) (153). DDR1 increases CXCL5 expression, which promotes the formation of NETs and infiltration of regulatory T cells (153). High DDR1 expression correlated with poor prognosis in breast cancer patients, and increased CXCL5 expression correlated with an increased number of malignant phenotypes of breast cancer cells (153). Li et al. (153) observed that DDR1/CXCL5 induces NETs formation to promote regulatory T cells immunoinfiltration, driving tumor growth and metastasis of breast cancer cells to the lungs.

Neutrophils from mammary tumor-bearing mice are more likely to form NETs than tumor-negative mice, and spontaneous NETosis has been associated with thrombosis in late-stage disease (154). Bacterial lung infection increases breast cancer metastasis in mice, due to the formation of NETs that capture circulating tumor cells (155). Yang et al. (156) demonstrated that tumor-associated aged neutrophils (Naged) have a greater ability to form NETs than non-aged neutrophils. Researchers found that Naged accumulated in the lung pre-metastatic niche early in the development of breast cancer in multiple mouse models, and were also found in the peripheral blood and lungs of metastatic breast cancer patients (156). Naged-generated NETs bound cancer cells but did not affect their proliferation or neutralize them (156). Naged persistently accumulates in the lung and generates NETs to capture cancer cells, thereby promoting lung metastasis of breast cancer (156). Mousset et al. (157) showed that chemotherapy associated with the treatment of lung metastases from breast cancer causes tumor cells to secrete IL-1β, which induces the NETs formation. NETs induce TGFβ-dependent EMT in cancer cells, which reduces the efficacy of therapy (157). Park et al. (123) observed that breast cancer cells can induce neutrophils to form NETs during metastasis. They also documented the presence of NETs in TNBC (123). The researchers’ results also suggest that cathepsin G is involved in the release of NETs (123). NETs induced by cancer cells affected the number of histologically detectable metastatic foci, which, according to the researchers, means that they can mediate the expansion of cancer cells (123).

Cai et al. (158) showed that the level of NETs in breast tumor tissues was higher compared to the expression of NETs in adjacent healthy breast tissues and correlated with the concentration of IL-8. In addition, they detected a higher recurrence rate in patients with higher NETs expression within the primary breast tumor, with a recurrence rate of 41.2% in the high NETs expression group compared to 3.6% in the low NETs expression group (158). Researchers also found higher concentrations of NETs in TNBC tissues (158). However, the results of the study by Martinez-Cannon et al. (159) showed that plasma levels of circulating NETs at diagnosis is not associated with recurrence in women with early stage breast cancer. The number of NETs generated varies depending on the breast cancer subtype, with the highest observed in TNBC (150). Zhao and Xie (160) showed that high expression of NETs-related genes in breast cancer patients correlates with better response to immunotherapy and a more favorable prognosis of the disease. NETs probably have a twofold effect on TNBC progression and the immune response associated with it (161). NET-related genes are highly expressed in TNBC and are associated with poor prognosis (161). The formation of NETs in the stroma of TNBC tissue is about two and a half times higher than in non-TNBC tissue (148, 161). NETs formation showed a positive correlation with tumor size, Ki67, and lymph node metastasis in patients with TNBC (148, 161). Inhibition of NETs reduces TNBC tumor growth and the formation of lung metastases (148, 161). More NETs were formed in the peripheral blood of patients with fever after TNBC-related surgery than in patients without postoperative fever (148). Neutrophils in the peripheral blood of patients with fever may therefore promote TNBC cell growth and invasion (148). In TNBC, reduced CD8+ T cell recruitment to the stroma was associated with poor clinical outcomes and unresponsiveness to immune checkpoint blockade (162). Expression of the cytokine Chi3l1 (Chitinase-3-like 1) was reduced in tumors lacking the transcription factor Stat3, which is commonly overactive in breast cancer and promotes an immunosuppressive tumor microenvironment (162). Chi3l1 is a biomarker of aggressiveness and breast cancer stage (162). CHI3L1 expression was elevated in human TNBC and other solid tumors showing T cells restriction (162). Chi3l1 also promoted neutrophil recruitment and NETs formation, which blocked T cells infiltration (162). A study by Rivera-Franco et al. (163) showed that NETs increases in proportion to the stage of the disease, and that higher levels of NE-DNA complexes are found in patients with breast tumor and local and distant metastasis compared to patients with tumor without metastasis. The concentration of circulating NETs was higher in patients with metastatic lung tumors than in patients with non-metastatic tumors (150).

Mesenchymal stromal cells recruit neutrophils to the lung and, through stimulation by complement component C3, are transformed into NETs (164). This process enabled the formation of a lung pre-metastatic niche for breast cancer cells (164). Researchers found higher levels of C3a in the serum of patients with metastases compared to the serum of patients with non-metastatic breast cancer and healthy women (164). Yang et al. (165) demonstrated that serum NETs could predict the occurrence of liver metastases in patients with early stage breast cancer. The mechanism was that excessive NETs could form in the livers of breast cancer patients before metastases could be detected and could facilitate the later development of liver metastases (165). One of the key components of NETs, ​​the DNA-histone complex, can recognize and bind to the transmembrane protein CCDC25 on breast cancer cells, thereby activating the ILK-β-parvin pathway to enhance tumor cell motility and lead tumor cells to form distant metastases (165). The researchers’ results also indicated that the NET-DNA complex induces cancer cell migration, adhesion, and proliferation through interaction with CCDC25 (165). CCDC25 expression was positively correlated with 3-hydroxy-3-methylglutaryl-CoAreductase (HMGCR) and citH3 expression in tissues from breast cancer patients (166). High CCDC25 and HMGCR expression was associated with poor prognosis in breast cancer patients (166).

Zhou et al. (167) showed that tumor-released autophagosomes (TRAP) induced NETs formation through activation of the neutrophil Toll-like receptor 4 (TLR4-Myd88-ERK/p38) signaling pathway mediated by HMGB1. Programmed death ligand 1 (PD-L1) carried by TRAP-induced NETs suppressed T cell function, thereby creating an immunosuppressive pre-metastatic environment that promoted breast cancer metastasis to the lungs (167). Circulating TRAP and NETs plasma levels in breast cancer patients with lung metastases were significantly higher compared to patients without metastases (167). Moreover, HMGB1 levels in circulating TRAPs correlates with NETs levels in peripheral blood and with lung metastases in breast cancer patients (167). These results indicate that the combination of TRAP, HMGB1 and NETs may serve as a potential biomarker for predicting breast cancer metastasis to the lungs, offering a new strategy for early detection and treatment of lung metastases in breast cancer patients (167). Long non-coding RNA (lncRNA) mediates NET-induced cancer cell metastasis in the TME, and may also alter interactions between tumor cells and the TME, leading to immunosuppression and treatment resistance, thereby enabling tumor cells to evade immune surveillance (168). Jiang et al. (168) constructed a prognostic model based on 10 lncRNAs associated with NETs, which showed good predictive ability and efficacy in breast cancer. This model was significantly correlated with the tumor immune microenvironment and anti-cancer treatments, indicating that these molecular changes may explain individual differences in treatment efficacy (168).

cfDNA has also been investigated as a biomarker for breast cancer (169). This parameter showed a good correlation with the stage of advancement and increased sensitivity to advanced disease (169). Kohler et al. (170) showed that both nuclear and mitochondrial free DNA have potential as biomarkers of breast tumors. However, circulating free nuclear DNA shows greater promise in terms of sensitivity and specificity (170). Similar studies were conducted by Mahmoud et al. (171), who also showed that extracellular nuclear and mitochondrial DNA levels were significantly higher in women with breast cancer compared to controls. The researchers also showed a significant association between parameter levels and histological grade, tumor stage, lymph nodes and hormone receptors (171). Li et al. (172) examined the expression levels of MMP-2 and MMP-9, a component of NETs, ​​in breast cancer tissues. The expression of both extracellular matrix metalloproteinases was correlated with lymph node metastases and tumor stage and influenced breast cancer prognosis (172). Akizuki et al. (173) showed that the concentration of immunoreactive NE in breast cancer tumors is an independent prognostic factor in patients undergoing radical surgery.

### 3.2 Ovarian cancer

Lee et al. (174) described that neutrophil influx into the omentum is a necessary step prior to metastasis, as studied in ovarian cancer models using the ID8 cell line. Tumor-produced inflammatory factors stimulated neutrophils to activate and produce NETs, ​​which promoted metastasis by trapping ovarian cancer cells (174). Researchers detected NETs in mice with ovarian tumors before metastasis and found that metastasis was reduced in PAD4-deficient mice (174).

Zhang et al. (175) investigated genes associated with NETs to predict the prognosis and assess drug sensitivity of ovarian cancer patients, based on bioinformatic analysis. In particular RAC2, one of the studied genes, was associated with NET formation and ovarian cancer metastasis (175). Lee et al. (174) detected NETs in clinical specimens of omental tissues from women with early-stage high-grade serous ovarian cancer (HGSOC). The researchers detected significantly more NE and protease-positive cells in diseased women than in omental tissues of healthy women (174). Muqaku et al. (176) also analyzed the involvement of NETs in HGSOC. The researchers’ results demonstrated a significant increase in NET components such as histones, MPO, MMP-9 and ELANE in ascites samples from patients with HGSOC (176).They also detected a correlation between metabolites associated with NETs formation, mainly NADPH oxidase-independent and eicosanoids (176). NETs formation in this tumor was associated with the release of S100A8/A9 protein (176). An increase of the S100A8/CRP ratio correlated with favorable survival of patients with HGSOC (176). Tomás-Pérez et al. (177) examined NETs-related biomarkers: cfDNA, nucleosomes, citH3, calprotectin and MPO in plasma and peritoneal fluid of patients with advanced-stage HGSOC. The researchers’ results indicate that HGSOC patients have higher levels of cfDNA, calprotectin and citH3 in plasma, while in peritoneal fluid they observed an increase in all biomarkers tested (177). This would suggest a possible involvement of NETs in advanced HGSOC and the possibility of using the above-mentioned markers in diagnostics, mainly cfDNA and calprotectin (177). Kim et al. (178) examined circulating NETs markers: histone-DNA complex, cfDNA and NE in the plasma of healthy women and patients with HGSOC. In patients with HGSOC, significantly higher levels of NETs markers were found compared to healthy patients (178). In patients with advanced HGSOC, the researchers observed higher levels of cfDNA compared to patients with early stage HGSOC (178). Ricciuti et al. (179) examined the serum and peritoneal fluid of patients with newly diagnosed ovarian cancer. The levels of genomic DNA (gDNA) they examined in serum before treatment, MPO, citH3 were associated with worse overall survival, while in peritoneal fluid, elevated factor H, a negative regulator of complement activation, was associated with improved overall survival of patients The results indicate the value of serum markers of cell damage, NETs and complement as potential prognostic biomarkers in patients with newly diagnosed epithelial ovarian cancer (179). The researchers also identified NE as an independent factor of poor prognosis for overall survival (178). Contrary results were obtained by Dobilas et al. (180) whose study showed that plasma levels of the citH3-DNA complex and double-stranded DNA (dsDNA) were not elevated in women with borderline or malignant ovarian tumors. NETs formation in early-stage ovarian cancer has been shown to be associated with serum IL-6 and G-CSF (181). The ability to form NETs in ovarian cancer may be stimulated by NE, VEGF, G-CSF and cytokines: TNFα, IL-2, IL-6, IL-17A in serum (181). Wang et al. (182) examined peripheral blood concentrations of citH3 and cfDNA in ovarian cancer patients and showed that they were elevated compared to controls (healthy individuals). citH3 showed a sensitivity of 0.8, specificity of 0.973, while cfDNA showed a sensitivity of 0.927, specificity of 0.947 (182). Measured together, the parameters had a sensitivity of 1.0 and specificity of 0.96 (182). Both citH3 and cfDNA levels were higher in patients with advanced disease compared to those with early stage disease (182).

cfDNA, which may originate from NETs, ​​is a marker for detecting ovarian cancer (183). Quantitative cfDNA analysis has unsatisfactory sensitivity but acceptable specificity for the diagnosis of ovarian cancer (183). cfDNA analysis in ovarian cancer can be used for early detection, disease monitoring, determining response to treatment and detection of minimal/molecular residual disease (MRD) and for identification of specific genetic alterations, such as BRCA1/2 mutations, that may be present in ovarian cancer (184). Elevated plasma concentrations of circulating nuclear and mitochondrial cfDNA have been found in patients with epithelial ovarian cancer (185). A study by Kalavska et al. (186) suggests that nuclear and mitochondrial cfDNA levels may be prognostic markers for ovarian cancer. Kamat et al. (187), determined preoperative total plasma cfDNA levels, which were found to be significantly elevated in patients with epithelial ovarian cancer, and that this level was an independent predictor of death from the disease. cfDNA showed independent prognostic significance in patients with multidrug-resistant ovarian cancer treated with bevacizumab (188). Singel et al. (189) demonstrated that mitochondrial DNA (mtDNA) from ascites of patients with epithelial ovarian cancer correlated with worse progression-free survival in advanced disease. Mitochondrial DAMPs activate neutrophils, which generate NETs (189). Mitochondrial and other DAMPs in ascites can activate neutrophils, which facilitate metastasis and block anti-tumor immunity (189). MMP-9 expression in ovarian cancers was significantly higher than in borderline and benign tumors (190).

### 3.3 Cervical cancer

A study by Fomenko et al. (191) showed that peripheral blood neutrophils generate NETs in 53.57% of cervical cancer patients studied before treatment. In healthy individuals, the researchers did not observe NETs, there was also no correlation between NETs formation and the stage of cervical cancer (191). The researchers also showed that the ability to form NETs varied after radiation therapy and that the addition of chemotherapeutic drugs to radiation therapy did not increase the percentage of NETs in the blood of cervical cancer patients, but stimulated the appearance of extracellular basophil traps (191). In cervical cancer, neutrophils are activated to form NETs (192). Yan et al. (192) found that increased NETs formation was an independent predictor of short recurrence-free survival in cervical cancer, and that combining NETs with the tumor, nodes, metastasis (TNM) classification system may improve prognosis of disease progression. Ning et al. (42) showed that neutrophilic infiltration and NETs formation were increased in cervical cancer patients with lymph node metastasis, which was confirmed in a mouse study, as well as a positive correlation between S100A7 expression and neutrophilic infiltration in this cancer. S100A7 protein plays a role in regulating cell migration, invasion, metastasis and EMT of cervical cancer (193). NETs have the ability to capture cervical cancer cells but had no cytotoxic effect on them, only the ability to promote lymph node metastasis (42). NETs increased the tumor migratory capacity by activating the P38-MAPK/ERK/NFκB pathway through interaction with TLR2 (42). NETs promoted lymphangiogenesis and increased lymphatic vessel permeability, thereby facilitating tumor cell movement (42). Cervical cancer-derived S100A7 showed a chemotactic effect on neutrophils and promoted NET generation by increasing ROS concentration (42).

### 3.4 Endometrial cancer

Endometrial/corpus uteri cancer is a gynecological cancer which ranks second and third, respectively in terms of new cases and deaths among gynecological malignancies (194, 195). Even with the advancement of diagnostics and modern treatment methods, it is often detected at a late stage and its prognosis is not favorable (195).

Seo et al. (196) determined the levels of circulating NETs markers: histone-DNA complex, double-stranded cell-free DNA (dsDNA) and neutrophil elastase in patients with endometrial cancer. The results of this study showed high levels of circulating NETs markers in patients with endometrial cancer (196). Abakumova et al. (197) showed that in the early stage of endometrial cancer, the ability of neutrophils to form NETs increased, but the number of cells captured by them decreased dramatically. As the disease progressed, the ability of neutrophils to form NETs increased, but the number of captured cells remained reduced (197). Researchers believe that enhanced generation of NETs with reduced killing function may promote tumor cell migration via neutrophil-tumor cell complexes (197). Ronchetti et al. (138) measured the levels of citH3, cfDNA, cfmtDNA, which are markers of NETs, in the serum of patients with endometrial cancer and using antibodies directed against citH3, NE, and histone 2B, examined NETosis in endometrial cancer tissues. The researchers showed the presence of NETosis in tissues from all stages of endometrial cancer differentiation, while in serum the markers were associated with the stage of the G1 and G2 differentiation stage of the tumor (138). They also showed a correlation between elevated cfDNA, citH3 levels and inflammatory features, which were examined through the number of lymphocytes, neutrophils, monocytes, platelets and fibrinogen levels (138). Endometrial cancer is closely associated with obesity, which is associated with increased neutrophil activation and increased generation of NETs, as shown in a study by D’Abbondanza et al. (115, 198).

Cicchillitti et al. (199) showed that cfDNA levels were higher in the serum of patients with G2 and G3 endometrial cancer compared to serum of patients with G1 endometrial cancer. Researchers also detected higher levels of cfDNA in the serum of patients with BMI>30 compared to the serum of patients with BMI<30 (199). Vizza et al. (200) in their study also observed a significant increase in cfDNA content in the serum of women with high-grade endometrial cancer compared to the serum of women with G1 endometrial cancer. High levels of cfDNA and detectable levels of tumor-derived DNA (ctDNA) in endometrial cancer patients are strong indicators of poor prognosis (201). Higher cfDNA levels were found in advanced stages of the disease (202).

### 3.5 Other corpus uteri cancers

Abakumova et al. (197) demonstrated that in patients with uterine fibroids the ability of neutrophils to capture cells via NETs was increased compared to healthy women.

### 3.6 Premature ovarian insufficiency

NETs have also been associated with POI (203). That’s because they can promote the release of cytokines that can damage tissues, cause inflammation, oxidative stress and fibrosis, factors potentially responsible for the pathogenesis of POI (203). Chen et al. (203) showed that vitamin D is involved in the development of premature ovarian failure by inhibiting the formation of NETs.

### 3.7 Cervicitis

Cervicitis leads to pelvic inflammatory disease (PID), endometritis, infertility, preterm birth and low birth weight (204). Liang et al. (205) identified genes associated with NETs using sequencing and machine learning techniques. The study identified five genes associated with inflammation and possibly cervicitis: PKM, ATG7, CTSG, RIPK3 and ENO1 (205).

### 3.8 Endometriosis

Berkes et al. (206) analyzed the presence of NETs in the peritoneal fluid of patients with endometriosis. Researchers have observed the release of NETs from neutrophils in patients with endometriosis and a small percentage of NETs in healthy patients (206). Quantification of NETs revealed a significantly higher number of NETs in patients with endometriosis compared to healthy patients (206). Most NETs were detected in patients with endometriosis stage I and II (206). These studies suggest that NETs may be involved in the complex and still being discovered endometriosis pathophysiology (206).

A study by Munrós et al. (207) showed significantly higher levels of NETs in patients with deeply infiltrating endometriosis compared to patients without surgical findings of endometriosis, which suggested that the presence of elevated plasma levels of circulating NETs may reflect the inflammatory state in this gynecologic disease. However, no differences in NETs levels were observed between patients with and without severe pelvic pain or between patients with and without infertility, regardless of the presence of endometrial lesions (207).

Zachariah et al. (208) showed that free nuclear DNA was significantly increased in women with endometriosis compared to healthy women. There was also a significant difference in circulating extracellular mitochondrial DNA levels between patients with endometriosis and patients with epithelial ovarian cancer (185).

### 3.9 Pregnancy

Pregnancy is associated with activation of circulating neutrophils, which may exhibit a pro-NETotic state (209). G-CSF, which increases during pregnancy, promotes NETs formation (209). Early in pregnancy, NETs formation is enhanced by chorionic gonadotropin, while in the perinatal period it is stimulated by estrogen (209). An interaction between estrogen and progesterone is formed, in which progesterone inhibits the formation of NETs (209). This means that extensive citrullination of histones is visible, but the complete formation of NETs is inhibited (209). Also, NE function is inhibited and regulated by progesterone (209).

### 3.10 Pregnancy-related diseases

It has been shown that neutrophils and NETs they form can play a role in pregnancy complications such as recurrent miscarriage, preterm labor or premature rupture of fetal membranes, gestational diabetes and preeclampsia (210).

Neutrophil recruitment, activation and release of NETs may be associated with excessive endothelial and placental damage (209). It appears that NETs may be involved in various stages of the reproductive cycle, starting with fertility and ending with fetal loss (211). The first suggestion that NETs might play a role in pregnancy-related disorders came from preeclampsia, where they were detected in large numbers in the intervillous space of the studied placentas (211).

Sur Chowdhury et al. (212) demonstrated the presence of material originating from NETs by detecting free DNA fragments complexed with MPO in the serum of healthy pregnant women, those with preeclampsia, and non-pregnant women. Free DNA/myeloperoxidase complexes derived from NETs were found in higher concentrations in the serum of healthy pregnant women than in non-pregnant women (212). This concentration increased gradually during pregnancy and was highest when preeclampsia occurred (212). In preeclampsia, endothelial dysfunction can be observed due to systemic inflammation involving neutrophils and NETs (213).

## 4 NETs and treatment options

Preventing NETs from forming or accelerating NETs degradation may be a potential therapeutic strategy (96). Several potential strategies can be considered, for example, treatment with deoxyribonuclease 1 (DNase I), which dissolves NETs or inhibition of PAD4 (214). Attempts to use NETs for treatment have been described in numerous studies and reviews (116, 215).

### 4.1 Breast cancer

Given that NETs formation stimulated invasion and migration of breast cancer cells, it seems a logical conclusion that inhibition of NETs formation or NETs digestion by DNase I blocks these processes (123). Treatment with DNase I-coated nanoparticles markedly reduced breast cancer metastasis to the lungs in mice (123). In a study by Park et al. (123), the PAD4 inhibitor Cl-amidine reduced NETs formation and blocked the ability of neutrophils to promote tumor invasion. In a study by Qi et al. (155), degradation of NETs by DNase I also significantly inhibited the formation of breast cancer metastases in the lungs.

To increase the efficacy of DNase-related therapy, since DNase is degraded quite rapidly under physiological conditions, Herre et al. (216) developed an adeno-associated virus (AAV) vector system to deliver mouse DNase I and tried it on a mouse model of metastatic breast cancer. The use of the AAV vector was aimed at prolonging DNAase viability (216). In addition to reduced breast cancer metastasis to the lungs in mice given the AAV-mDNase I (adeno-associated virus vector system for delivery of murine DNase I), they also observed a lower value of the renal hypoperfusion biomarker, neutrophil gelatinase-associated lipocalin (NGAL), in these mice compared to mice that received DNase without the vector (216).

Sivelestat, an NE inhibitor, has been investigated for use in the treatment of breast cancer, specifically epithelial growth factor receptor 2 (HER2) positive breast cancer (217). NE interacts with tumor growth factor-α (TGF-α), which is present in breast cancer cells, and inhibiting this interaction would adversely affect tumor cell proliferation (217). Nawa et al. (217) showed that the combined use of sivelestat and trastuzumab inhibited cell proliferation more intensively than with either drug alone.

Zhu et al. (152) found that the NF-κB essential modifier-binding domain (NBD) peptide reduced IL-8 levels and NETs formation, resulting in inhibition of primary tumor growth, inhibition of lung metastasis in mouse models of human breast cancer and in a mouse model of spontaneous breast cancer. Also, inhibition of NETs production by the PAD4 inhibitor reduced NF-κB activation, resulting in reduced metastasis (152).

Tang et al. (166) showed that affecting cholesterol biosynthesis could be a therapeutic strategy for breast cancer. This is because cholesterol biosynthesis induced by ASPP2 depletion in mouse breast cancer cells and human breast cancer cell cultures promoted the formation of NETs *in vitro*, as well as in breast cancer metastasis to the lungs in ASPP2-deficient mice (166)Cholesterol biosynthesis is also a positive regulator of CCDC25 expression, and increased CCDC25 expression is associated with breast cancer metastasis (166). Simvastatin and berberine, inhibitors of cholesterol synthesis, effectively blocked NETs formation induced by ASPP2 depletion, which may have therapeutic effects on breast cancer metastasis (166).

Yu et al. (218) examined resveratrol (RES), a polyphenolic natural phytoalexin and silent information regulator-1 (SIRT1) agonist, which inhibited NETs formation after CTSC treatment. In *in vivo* studies, RES impeded the formation of breast cancer metastases in a mouse model of breast cancer (218). Also, serum levels of NETs markers, MPO-DNA and NE-DNA in the mouse model of breast cancer were significantly lower after treatment (218). RES, among other things, inhibits histone H3 citrullination, which is essential for NETs formation (218). The researchers also found that NETs were suppressed by RES in bone marrow neutrophils after CTSC treatment, while specific SIRT1 deficiency in neutrophils promoted their formation, and thus breast cancer metastasis to the lungs (218).

Zeng et al. (219) studied kaempferol, a flavonoid, and found that it had an inhibitory effect on primary tumor growth and lung metastasis in a mouse model of breast tumor. After treating lung metastases with the compound, they also observed reduced expression of citH3, a biomarker of NETs (219). The researchers also found that kemferol is specific for NETs, with no effect on neutrophils (219).

Lu et al. (220) developed a micellar nanoparticle of low-molecular-weight heparin and astaxanthin (LMWH-AST/DOX, LA/DOX NP) loaded with doxorubicin, which has the ability, among other things, to reduce the recruitment of neutrophils in the liver and myeloid-derived suppressor cells (MDSCs) in the lung and tumor by blocking P-selectin. The nanoparticle has the ability to inhibit the formation of NETs, thereby inhibiting breast cancer metastasis to the lung and liver (220).

Zhao et al. (221) examined the effects of dihydrotanshinone I (DHT), a compound derived from *Salvia miltiorrhiza Bunge* (*S. miltiorrhiza*) on breast cancer. In their study, DHT inhibited the formation of NETs and attenuated breast cancer metastasis to the lungs induced by NETs (221).

### 4.2 Ovarian cancer

Metastasis to the omentum, a common occurrence in ovarian cancer, was reduced in mice deficient in neutrophil-specific PAD4 (174). Blocking NETs formation with a pharmacological PAD4 inhibitor also reduced omentum colonization (174).

Doxorubicin (DOX), used in the treatment of ovarian cancer, is captured by NETs, preventing the substance’s therapeutic effect of inducing apoptosis of tumor cells (222). Tamura et al. (222) demonstrated that the reduced diffusion of the drug was restored after degradation of NETs by DNAase I.

### 4.3 Cervical cancer

Ning et al. (42) demonstrated that digestion of NETs with DNAase 1 or inhibition of TLR2 with chloroquine eliminated the metastatic potential of cervical cancer, as observed by reduced metastasis to inguinal lymph nodes.

No studies combining the inhibition of NETs formation and endometrial cancer have been conducted to date.

## 5 Conclusions

Initially, numerous observations indicated undoubtedly positive aspects of NETs formation, however, as it results from the studies conducted so far, their formation may also accompany the pathogenesis of many diseases, including diseases of the female reproductive organs, in which excessive or chronic NETs formation or their improper/abnormal removal has been demonstrated. Moreover, the conducted studies do not fully explain the causes of interactions between cancer cells and NETs, which may prove helpful in explaining and understanding many aspects of the body’s immune response against cancer cells, as well as in developing new diagnostic and therapeutic strategies in patients with breast cancer and gynecological cancers. The few studies on the role of NETs in the course of other reproductive organ diseases also indicate their participation in the pathogenesis of these diseases, which requires further, more detailed research taking into account the importance of NETs as potential biomarkers and their use in therapy.
