# Supplementary material for: Neutrophil extracellular traps in diseases of the female reproductive organs
Source: Front Immunol. 2025 May 5;16:1589329. doi: 10.3389/fimmu.2025.1589329 (PMC12086147; doi:10.3389/fimmu.2025.1589329)
Supplement: Supplementary file 1 [file Table1.docx]

| **DRUG** | **MECHANISM** | **EFFECT** | **REFERENCE** |
| --- | --- | --- | --- |
| **BREAST CANCER** | | | |
| DNase I-coated nanoparticles | NETs digestion | reduced lung metastases in mice | [116] |
| DNase I | NETs degradation | suppresses metastasis in lung | [153] |
| AAV-mouse DNase I  (adeno-associated virus vector system for delivery of murine DNase I) | NETs degradation | reduce cancer-associated impairment of renal function and development of metastasis | [227] |
| PAD4 inhibitor | blocking NETs formation | preventing metastasis | [116] |
| simvastatin and berberine | cholesterol synthesis inhibitors, blocking NET formation | blocking cholesterol promoted CCDC25 expression, enhanced in breast cancer and metastasis | [164] |
| sivelestat and trastuzumab | NE inhibitor | suppressed cell proliferation | [228] |
| resveratrol | inhibition of citrullination of histones H3, which is essential for chromatin decondensation and NETs formation | prevent neutrophil cell death and breast cancer metastasis | [229] |
| NBD peptide + PAD4 inhibitor | selective inhibition of NF-κB and PAD4-dependent NETs formation | decreased NF-κB activation and tumor metastasis, reduce IL-8 level and NETs formation, suppressed primary tumor growth and/or lung metastasis in human breast cancer mouse xenograft models and mouse spontaneous breast cancer model | [150] |
| kaempferol | suppressed ROS production, anti-NETs effects | suppressed primary tumour growth and lung metastasis in mouse breast tumour model | [230] |
| doxorubicin-loaded micellar low-molecular-weight-heparin-astaxanthin nanoparticle (LMWH-AST/DOX, LA/DOX NP) | inhibiting the formation of NETs | inhibition of liver and lung metastasis, alleviation of the inflammatory and immunosuppressive TME | [231] |
| dihydrotanshinone I (DHT) | suppressing neutrophil infiltration and NETs formation | inhibition of lung metastasis | [232] |
| **OVARIAN CANCER** | | | |
| DOX + DNase I | NETs digestion | augmented response of ovarian cancer to DOX | [233] |
| PAD4 inhibitor | blocking NETs formation | decreased omental colonization | [177] |
| **CERVICAL CANCER** | | | |
| DNase 1 | NETs digestion | elimination of NETs-induced metastatic potential | [197] |
| chloroquine | inhibiting TLR2 | hindering NETs-induced metastatic potential | [197] |
