# Supplementary material for: Neutrophil extracellular traps in diseases of the female reproductive organs
Source: Front Immunol. 2025 May 5;16:1589329. doi: 10.3389/fimmu.2025.1589329 (PMC12086147; doi:10.3389/fimmu.2025.1589329)
Supplement: Supplementary file 2 [file Table2.docx]

| **MATERIAL** | | **RESEARCH** | **REFERENCE** |
| --- | --- | --- | --- |
| **TISSUE RESEARCH** | | - NETs are found in tissues of all grades of endometrial cancer | [129] |
| **RESEARCH ON PLASMA** | **NETs markers** | - high concentrations of histone-DNA complex, dsDNA and NE in patients with endometrial cancer | [203] |
|  | **NETs components** | - higher cfDNA concentrations in advanced disease stages | [204] |
|  |  | - high cfDNA levels and detectable ctDNA in patients with endometrial cancer are strong indicators of poor prognosis | [205] |
| **RESEARCH ON SERUM** | **NETs** | - NETs can promote cancer cell migration by forming neutrophil-tumor cell complexes | [206] |
|  | **NETs markers** | - citH3, cfDNA, free mitochondrial DNA (cfmtDNA) levels were associated with G2 and G3 endometrial cancer - there is a correlation between elevated cfDNA, citH3 levels and inflammatory features | [129] |
|  | **NETs components** | - higher cfDNA concentration in patients with G2 and G3 endometrial cancer compared to patients with G1 endometrial cancer - higher cfDNA concentration in patients with BMI>30 compared to patients with BMI<30 | [207] |
|  |  | - higher cfDNA levels are found in women with high-grade endometrial cancer compared to women with G1 endometrial cancer | [208] |
