# Supplementary material for: Neutrophil extracellular traps in diseases of the female reproductive organs
Source: Front Immunol. 2025 May 5;16:1589329. doi: 10.3389/fimmu.2025.1589329 (PMC12086147; doi:10.3389/fimmu.2025.1589329)
Supplement: Supplementary file 3 [file Table3.docx]

| **MATERIAL** | | **RESEARCH** | **REFERENCE** |
| --- | --- | --- | --- |
| **RESEARCH ON CELL LINES** | | - NETs have the ability to capture cervical cancer cells and promote metastasis to lymph nodes - NETs promoted lymphangiogenesis and increased lymphatic vessel permeability, facilitating cancer cells movement - NETs increased tumor migration capacity by activating the P38-MAPK/ERK/NFκB pathway via interaction with TLR2 - there is a positive correlation between S100A7 expression, neutrophil infiltration and cervical cancer - cervical cancer-derived S100A7 had a chemotactic effect on neutrophils and promoted NETs generation by increasing ROS concentration | [197] |
| **RESEARCH ON ANIMALS** | | - more significant lymph node metastases were found in “NETs” mice | [197] |
| **TISSUE RESEARCH** | | - increased NETs formation was an independent prognostic factor for short recurrence-free survival - combining NETs with TNM classification system may improve disease prognosis | [198] |
|  |  | - NETs formation was increased in patients with lymph node metastases | [197] |
| **RESEARCH ON PERIPHERAL BLOOD** | **NETs** | - in 53.57% of the examined patients before treatment neutrophils generate NETs - in healthy individuals, researchers did not observe NETs - no relationship between NET development and the stage of cervical cancer - the ability to form NETs varied after radiotherapy - adding chemotherapy to radiotherapy did not increase the rate of NETs in patients with cervical cancer | [199] |
|  |  | - neutrophil infiltration and NETs formation were increased in patients with cervical cancer with lymph node metastases | [197] |
