# Supplementary material for: Neutrophil extracellular traps in diseases of the female reproductive organs
Source: Front Immunol. 2025 May 5;16:1589329. doi: 10.3389/fimmu.2025.1589329 (PMC12086147; doi:10.3389/fimmu.2025.1589329)
Supplement: Supplementary file 4 [file Table4.docx]

| **MATERIAL** | | **RESEARCH** | **REFERENCE** |
| --- | --- | --- | --- |
| **RESEARCH ON CELL LINES** | | - NETs promoted metastasis by binding to ovarian cancer cells | [177] |
| **RESEARCH ON ANIMALS** | | - metastasis was reduced in PAD4-deficient ovarian tumor-bearing mice | [177] |
| **GENE RESEARCH** | | - RAC2, a NET-related gene, has been linked to NETs formation and metastasis in ovarian cancer | [178] |
| **TISSUE RESEARCH** | | - significantly more NE and protease positive cells are found in sick women omental tissues than in the omental tissues of healthy women | [177] |
|  |  | - MMP-9 expression in ovarian cancer was higher than in borderline and benign tumors | [179] |
| **ASCITES RESEARCH** | | - significant increase in histones, MPO, MMP-9 and ELANE occurs in patients with HGSOC - there is a correlation between metabolites associated with the NETs formation and eicosanoids - NETs formation has been associated with the release of S100A8/A9 protein - increased S100A8/CRP correlated with favorable survival in patients with HGSOC | [180] |
|  |  | - mtDNA in patients with epithelial ovarian cancer correlated with poor progression-free survival in advanced disease | [181] |
| **PERITONEAL FLUID RESEARCH** | | - in patients with HGSOC, an increase in cfDNA, nucleosomes, citH3, calprotectin and MPO was observed | [182] |
| **RESEARCH ON PLASMA** | **NETs markers** | - patients with HGSOC have higher levels of cfDNA, calprotectin and citH3 | [182] |
|  |  | - in patients with HGSOC, higher concentrations of histone-DNA complex, cfDNA and NE were found compared to healthy patients - higher concentrations of cfDNA are found in patients with advanced HGSOC compared to patients with early stage HGSOC - NE is an independent factor of poor prognosis for overall survival | [183] |
|  |  | - citH3-DNA complex and dsDNA levels were not elevated in women with borderline or malignant ovarian tumors | [184] |
|  | **NETs components** | - cfDNA can be used for early detection, disease monitoring, treatment response and minimal residual diesease (MRD) detection, and identification of specific genetic changes | [185] |
|  |  | - increased levels of circulating extracellular nuclear and mitochondrial DNA are found in patients with epithelial ovarian cancer | [186] |
|  |  | - cfDNA of nuclear and mitochondrial origin concentrations may be prognostic markers for ovarian cancer | [187] |
|  |  | - preoperative total cfDNA levels are significantly elevated in patients with epithelial ovarian cancer and were an independent predictor of disease-related death | [188] |
|  |  | - cfDNA showed independent prognostic significance in patients with multidrug-resistant ovarian cancer treated with bevacizumab | [189] |
| **RESEARCH ON PERIPHERAL BLOOD** | **NETs markers** | - citH3 and cfDNA levels in ovarian cancer patients were increased compared to healthy controls - the concentrations of both parameters were higher in patients with an advanced stage of the disease compared to patients with an early stage | [111] |
| **RESEARCH ON SERUM** | **NETs markers** | - in early stage ovarian cancer, NETs formation is associated with IL-6 and G-CSF - NETs formation can be stimulated by NE, VEGF, G-CSF, TNFα, interleukin-2 (IL-2), IL-6, interleukin-17A (IL-17A) | [190] |
