# Supplementary material for: Neutrophil extracellular traps in diseases of the female reproductive organs
Source: Front Immunol. 2025 May 5;16:1589329. doi: 10.3389/fimmu.2025.1589329 (PMC12086147; doi:10.3389/fimmu.2025.1589329)
Supplement: Supplementary file 5 [file Table5.docx]

| **MATERIAL** | | **RESEARCH** | **REFERENCE** |
| --- | --- | --- | --- |
| **RESEARCH ON CELL LINES** | **NETs** | - NETs activate tumor growth and promote metastasis from breast to lung | [148] |
|  |  | - NETs regulate the gene expression of factors associated with the pro-inflammatory and pro-metastatic properties of breast cancer cells: IL-1β, IL-6, IL-8, CXCR1, matrix metalloproteinase-2 (MMP-2), MMP-9 and CD44 - NETs contribute to the acquisition of metastatic properties during breast cancer progression | [149] |
|  |  | - cancer cell-derived factors: IL-8 and GCS-F stimulate neutrophils to form NETs - NETs promote breast cancer cell progression | [150] |
|  | **NETs components** | - MMP-9 expression was correlated with lymph node metastases, tumor stage and influenced disease prognosis | [151] |
| **RESEARCH ON ANIMALS** | | - NETosis has been associated with thrombosis in mammary tumor–bearing mice at late stages of the disease | [152] |
|  |  | - NETs capture circulating tumor cells that cause lung metastases | [153] |
|  |  | - Naged accumulates in the lungs and produces NETs, ​​capturing cancer cells, which promotes lung metastases | [154] |
|  |  | - chemotherapy causes cancer cells to secrete IL-1β, which induces the formation of NETs - NETs induce TGFβ-dependent EMT in cancer cells, which reduces the efficacy of therapy | [155] |
|  |  | - mesenchymal stromal cells recruit neutrophils to the lungs and, through stimulation by complement component C3, form NETs | [156] |
|  |  | - breast cancer cells can induce neutrophils to form NETs during metastasis - NETs are present in TNBC - cathepsin G is involved in the release of NETs - NETs induced by cancer cells influenced the number of histologically detectable metastatic foci | [116] |
|  |  | - DNA-histone complex can recognize and bind to the transmembrane protein CCDC25 on breast cancer cells, thereby activating the downstream ILK-β-parvin pathway to increase tumor cell motility and lead to the formation of distant metastases | [157] |
|  |  | - collagen increases DDR1 expression, which increases CXCL5 expression, which promotes NETs formation and regulatory T cell infiltration, drives tumor growth and lung metastasis - high DDR1 expression correlated with poor prognosis | [158] |
| **GENE RESEARCH** | | - high expression of NET-related genes correlates with better response to immunotherapy and more favorable disease prognosis | [159] |
|  |  | - NET-related lncRNAs showed good predictive ability and efficacy in breast cancer diagnosis | [160] |
| **TISSUE RESEARCH** | **NETs** | - the content of NETs in breast tumor tissues was higher compared to adjacent normal breast tissues - NETs expression correlated with IL-8 concentration - a higher frequency of recurrence occurred in patients with higher expression of NETs in the primary breast tumor | [161] |
|  |  | - the highest number of NETs was observed in TNBC - circulating NETs concentration was higher in patients with metastatic lung tumors than in patients with non-metastatic tumors | [148] |
|  |  | - NET-related genes are highly expressed in TNBC and are associated with poor prognosis - NETs formation in the stroma of TNBC tissue is about two and a half times higher than in non-TNBC tissue - NETs showed positive correlation with tumor size, Ki-67 and lymph node metastasis in TNBC patients - NET inhibition effectively suppresses TNBC tumor growth and lung metastasis | [138,162] |
|  |  | - in TNBC, limited CD8+ T cell influx into the stroma was associated with unfavorable clinical outcomes and lack of response to immune checkpoint blockade - expression of the cytokine Chi3l1 was reduced in tumors lacking the transcription factor Stat3 - CHI3L1 expression was elevated in TNBC and other solid tumors exhibiting T cell restriction - Chi3l1 promoted neutrophil recruitment and NETs formation, which blocked T cell infiltration | [163] |
|  |  | - CCDC25 expression was positively correlated with HMGCR and citH3 expression in breast cancer tissues - high expression of CCDC25 and HMGCR was associated with poor prognosis | [164] |
|  | **NETs components** | - immunoreactive NE concentration is an independent prognostic factor in patients undergoing radical surgery | [165] |
| **RESEARCH ON PLASMA** | **NETs** | - circulating NETs concentration at diagnosis is not associated with recurrence in women with early stage breast cancer | [166] |
|  |  | - circulating TRAP and NETs concentrations in breast cancer patients with lung metastases were higher compared to patients without metastases | [167] |
|  | **NETs markers** | - NETs concentration increases in proportion to the stage of the disease - higher concentrations of NE-DNA complexes are found in patients with breast tumors with local and distant metastases compared to patients with tumors without metastases | [168] |
|  | **NETs components** | - nuclear and mitochondrial cfDNA have potential as biomarkers for breast tumors | [169] |
| **RESEARCH ON PERIPHERAL BLOOD** | **NETs** | - patients with fever following TNBC-related surgery developed more NETs than patients without postoperative fever | [138] |
|  |  | - HMGB1 concentration in circulating TRAPs correlates with NETs concentration in peripheral blood and with lung metastases | [167] |
|  | **NETs components** | - nuclear and mitochondrial DNA concentration in women with breast cancer was higher compared to the control group - there is a correlation between the parameters and histological grade, tumor stage, lymph nodes and hormone receptors | [170] |
| **RESEARCH ON SERUM** | **NETs** | - NETs in serum may predict the occurrence of liver metastases in patients with early-stage breast cancer | [157] |
